# Drug re-purposing to improve outcomes in the management of prostate cancer – aims, outcome measures and design of current phase III trials

**DOI:** 10.1186/s40360-025-01077-w

**Published:** 2026-01-24

**Authors:** Duncan C. Gilbert, Ruth E. Langley, Dami Ayadi, Mannab Berhanu, Lakshmi Kowdley Hemanth, Seunghee Kwon, Hossameldin Abdallah, Angela Meade, Noel Clarke, Silke Gillessen, Nicholas James, Gauthier Bouche, Mahesh Parmar, Matthew Nankivell, Laura Murphy

**Affiliations:** 1 MRC Clinical Trials Unit at UCL, Institute of Clinical Trials and Methodology, 90 High Holborn, London, UK; 2 Division of Cancer Sciences, University of Manchester, Manchester, UK; 3 The Christie Hospital NHS Foundation Trust, Manchester, UK; 4 Department of Urology, Salford Royal Hospital, Manchester, UK; 5 Institute of Oncology of Southern Switzerland, EOC, Bellinzona, Switzerland; 6 Faculty of Biomedical Sciences, Università della Svizzera Italiana, Lugano, Switzerland; 7 Swiss Group for Clinical Cancer Research (SAKK), Effingerstrasse 33, Bern, CH-3008 Switzerland; 8 The Institute of Cancer Research and The Royal Marsden Hospital, London, UK; 9 The Anticancer Fund, Brusselsesteenweg 11, Meise, 1860 Belgium

**Keywords:** Prostate cancer, Repurposing, Phase III randomised controlled trials

## Abstract

Prostate cancer remains a major cause of cancer morbidity and mortality and is rising in incidence across the world. Although a succession of randomised controlled trials have improved outcomes across all stages of disease, there remain significant clinical challenges and unmet needs. Repurposed drugs have attracted interest in the treatment of prostate cancer, where a number of agents have been proposed with a range of mechanisms of action suggesting potential benefits. A major advantage of such drugs is the wealth of pre-existing patient safety data typically available, meaning repurposed agents would be expected to have a known and often low toxicity profile and also (as often late in their developmental pathway and hence generic drugs are available) attractive in terms of cost effectiveness. The evidence required for repurposed drugs to become accepted standards of care and enter the treatment formulary however is no less arduous than conventional development pathways, where confirmatory results from randomised phase III trials remain the absolute requirement. Additionally, these efforts are often led by academic trial groups – with challenges then in the steps to licensing that are typically undertaken by industry. To date, despite large scale initiatives, there is a paucity of drugs successfully repurposed in this way. Using examples across the different clinical scenarios we discuss the opportunities and challenges, design and analyses of current phase III trials testing repurposed drugs for the treatment of patients with prostate cancer and highlight where future success may come.

## Clinical trial number

Not applicable.

## Background

Prostate cancer remains a major cause of morbidity and mortality in men, responsible for 55,000 new cases and 12,000 deaths in the UK per annum [1]. It is projected to rise in incidence from 1.4 million cases in 2020 to 2.9 million worldwide in 2040 as a result of changing demographics and improving life expectancy [2].

Until the turn of the last century, treatment typically consisted of surgical excision (radical prostatectomy) for the earliest, localised cases and androgen deprivation therapy (ADT) for the rest of patients, with the addition of radiotherapy for men without overt evidence of metastatic disease [2]. Over the last 20 years, several key developments have delineated different clinical stages of disease while clinical trials have defined corresponding standards of care that have seen outcomes improve dramatically.

The availability of Prostate Specific Antigen (PSA) testing significantly increased the detection of early disease, but until recently the low rates of specificity for clinically significant disease made over-diagnosis a challenge and undermined its use in population screening. However, the addition of multi-parametric MRI in the pathway for men with early (PSA-based) detection can reliably exclude clinically insignificant cancers without the need for biopsy and this, together with targeted PSA testing in high-risk populations, seems likely to be adopted in high-income countries [3, 4].

Advances in treating localised, intermediate risk disease have improved outcomes, with refinements in surgical techniques - progressing from laparoscopic to robotic-assisted radical prostatectomy – reducing operative complications and hospital stay. Radiotherapy schedules have also been optimised where sequential large-scale, phase III trials have taken the previous standard of care (74 Gy external beam radiotherapy in 37 fractions (#) over 7.5 weeks [5]) through moderate hypofractionation (60 Gy in 20# over four weeks [6]) to stereotactic radiotherapy techniques that treat the prostate in 5# [7]. Additionally the benefit of the addition of radiotherapy to 2–3 years of ADT in patients with locally advanced but non-metastatic (M0) disease was demonstrated and this is now a standard of care [8, 9].

The efficacy of systemic agents in the treatment of patients with prostate cancer has typically been demonstrated firstly in patients with metastatic, castrate-resistant disease and then in the ‘upfront’ setting before patients have experienced progression on ADT. In this way the chemotherapy agent docetaxel was shown to improve survival in patients progressing on ADT and subsequently in patients commencing ADT for metastatic disease in the CHAARTED and STAMPEDE trials [10–12]. A raft of androgen receptor pathway inhibitors (ARPI) followed suit, including abiraterone acetate, enzalutamide, apalutamide and darolutamide, and these are now typically considered standard of care in newly diagnosed, hormone sensitive patients [13–17] presenting with locally advanced (abiraterone) or metastatic (all) disease. The second-generation taxane, cabazitaxel, has efficacy in patients previously treated with Docetaxel [18]. There is increasing interest in more targeted approaches. This includes radiopharmaceuticals – where initially radium 223 demonstrated benefit in patients with extensive bone metastases, and more recently Prostate Specific Membrane Antigen (PSMA)-Lutetium offers metastases directed therapy [19, 20]. Molecularly targeted agents include Poly ADP ribose polymerase (PARP) inhibitors in patients with DNA damage repair defects and potentially immune checkpoint inhibitors for the small number of patients whose tumours harbour deficiencies in mis-match repair (dMMR) [21].

**Fig. 1:**
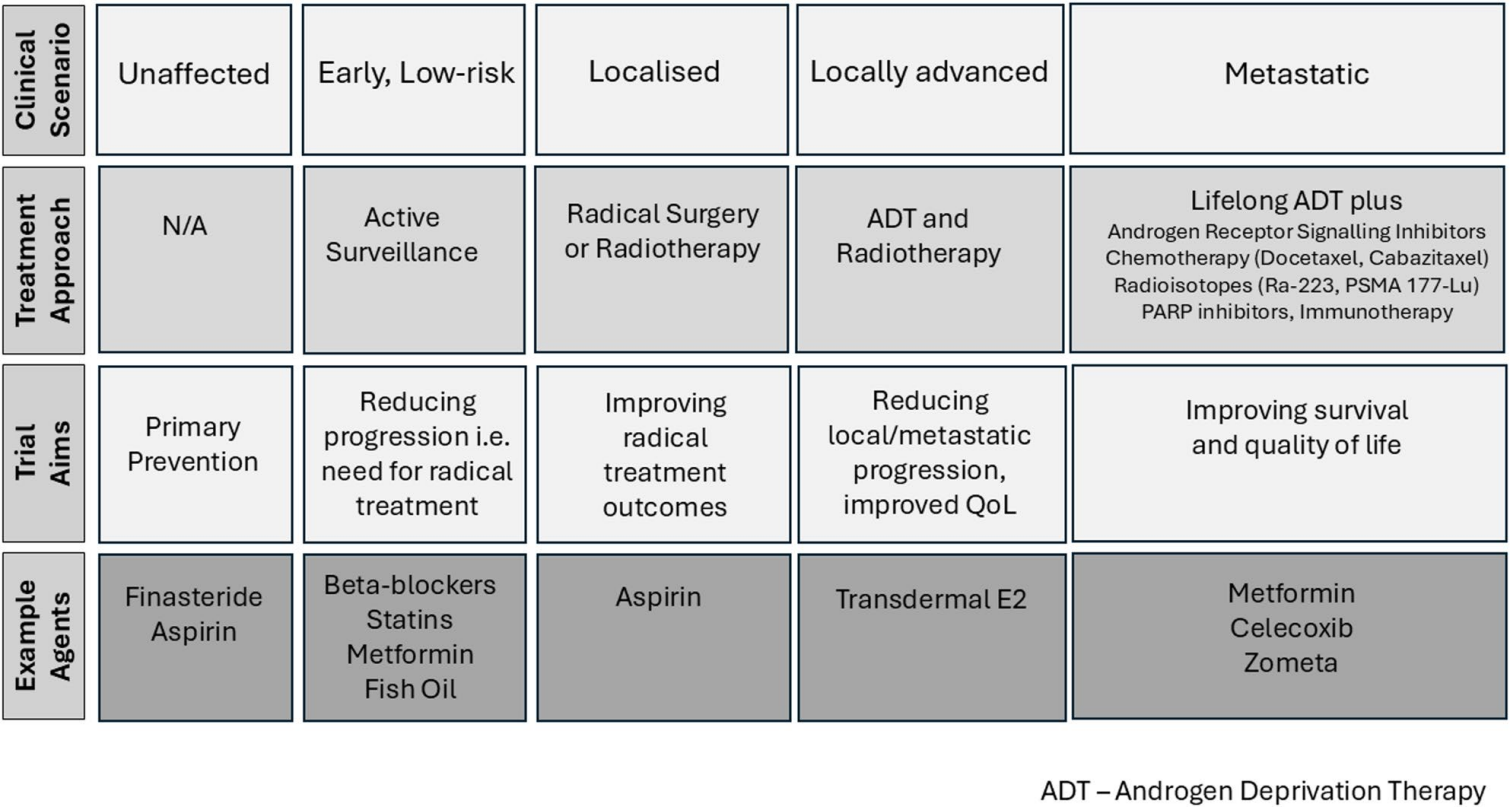
Clinical scenarios affecting patients with prostate cancer and opportunities to improve outcomes

Despite this progress there still exist specific clinical areas with open questions, namely primary prevention, patients undergoing active surveillance where treatments might reduce the requirement for active intervention, improving outcomes after radical treatment, mitigating the side-effects of ADT, and improving outcomes for those patients who still develop locally advanced or metastatic disease (Fig. 1). While new targets and corresponding drugs continue to emerge, the global burden of prostate cancer makes it not just an opportunity but a necessity to assess currently licensed medicines for their potential benefits in this population. A range of different approaches and mechanisms have been suggested (reviewed by Turanli and colleagues) [22]. To this end we use a series of current phase III trials testing repurposed medicines, many of which we are directly involved with, to demonstrate this strategy and discuss methods taken to investigate this approach in men with prostate cancer (Table 1).

**Table 1:** Examples of phase III randomised controlled trials investigating repurposed drugs for the treatment of different stages of prostate cancer highlighting trial design features in different clinical scenarios

Clinical Scenario	Primary Outcome Measure	Repurposing Trial and Agent	Design Features
Prevention	Prostate Cancer Incidence	PCPT - Finasteride	Primary outcome of development of histologically proven prostate cancer, targeting 25% reduction in incident cancers, 92% power, two sided alpha 0.05.
Active Surveillance	Prostate Cancer Progression / Time to Radical Treatment	MAST (NCT01864096) - Metformin	Primary outcome of pathological progression or time to radical treatment (surgery/androgen deprivation/radiotherapy), powered to show superiority.
Radical Treatment	Biochemical Recurrence Free Survival	ADD ASPIRIN (NCT02804815) - Aspirin	Primary outcome biochemical recurrence free-survival: given the very long lead time and uncertain association between recurrence after radical treatment and metastases/death this was considered optimal by trial group with patient input.
Long term ADT	Metastases-Free Survival	PATCH (NCT00303784) – transdermal Estradiol	Non-inferiority design for primary (oncological) outcome, secondary outcome measures include major non-oncological morbidity (e.g. cardiovascular events) and quality of life to assess benefits in event non-inferior for cancer treatment.
Locally advanced/metastatic disease	Metastases-Free Survival (M0) / Overall Survival (M1)	STAMPEDE (NCT00268476) – Zoledronic Acid, Celecoxib, Metformin	Metastases-Free Survival is a validated surrogate outcome for overall survival in the context of locally advanced prostate cancer and allows an earlier answer / realistic sample size to influence standards of care. Overall survival remains the key outcome in patients with metastatic disease. STAMPEDE is powered for superiority of these outcome measures.

## Primary prevention of prostate cancer

As a major health challenge, prevention of prostate cancer could have multiple benefits. Given that drugs in this space are intended for individuals without cancer but at high risk, and potentially administered over many years, safety is of utmost importance. The established long-term safety profiles from repurposed agents make them particularly well-suited for preventive interventions. Various classes of drugs have been proposed or investigated for their potential role in prostate cancer prevention based on their proposed mechanism of action, primarily through hormonal modulation, anti-inflammatory effect and metabolic modulation.

Androgens play a key role in the development and progression of prostate cancer. The Prostate Cancer Prevention Trial (PCPT) evaluated finasteride, a 5ɑ-reductase inhibitor, in a preventative setting [23]. This is analogous to the repurposing of the aromatase inhibitor Anastrozole for the prevention of breast cancer in women at high risk of developing the disease [24]. PCPT was designed to have 92% power to detect a 25% reduction in prevalence of biopsy-proven prostate cancer using a two-sided test with α = 0.05 (Table 1). 18,880 men were randomised to finasteride vs. placebo, and with 18 years of follow up, prostate cancer was diagnosed in 989 of 9423 (10.5%) in the finasteride group and 1412 of 9457 (14.9%) in the placebo group (relative risk in the finasteride group, 0.70; 95% confidence interval [CI], 0.65 to 0.76; *P* < 0.001). Interestingly high-grade disease was diagnosed more frequently in the finasteride arm. 15-year survival was 78.0% in men randomised to finasteride and 78.2% placebo (hazard ratio for death 1.02 (95% CI, 0.97 to 1.08; *P* = 0.46) [23]. Even at 18 years follow up, there was no significant difference in overall survival or survival after a prostate cancer diagnosis between the finasteride and placebo groups highlighting the complex relationship between prostate cancer incidence and ultimate impact on survival [25].

Aspirin has been suggested to affect multiple mechanisms that could confer an anti-cancer effect including immunomodulation, cell metabolism, gene repair, inflammation reduction, platelet activation, and gut microbiota improvement [26]. Recent work has begun to elucidate a mechanism whereby, through the inhibition of cyclo-oxygenase 1 (COX1) in platelets, aspirin reduces the production of thromboxane A2 (TXA_2_) [27]. In pre-clinical models the abolition of TXA_2_ signalling reduces metastatic dissemination [28, 29] and perhaps most strikingly inhibition of platelet derived TXA_2_ has been shown to enhance T-cell immune mediated clearance of metastases in mouse models [30].

The strongest data supporting a role for aspirin in cancer prevention comes from randomised studies investigating aspirin to prevent cancers in patients with Lynch syndrome [31]. Prior data with respect to long-term outcomes from patients randomised in trials testing aspirin in the context of cardiovascular disease, showed aspirin use was associated with a lower risk of cancer-related death [32]. With respect to primary prevention in particular [33], aspirin reduced the risk of a cancer diagnosis (hazard ratio 0.88 (95%CI 0.80–0.98, *P* = 0.017) with the effect proportional to time on treatment. Overall, pooled data from case–control and cohort studies suggest that aspirin reduces the risk of a diagnosis of several common cancers including prostate cancer (relative risk 0.90, 0.85–0.96, *P* < 0.001) [33]. One piece of contrasting data is the results from the ASPREE trial (Aspirin in Reducing Events in the Elderly (NCT01038583)) where cancer-related deaths were actually higher in the aspirin group [34]. With a relatively small effect size, a primary prevention study for prostate cancer alone would likely be prohibitively large – whether this might be included as a composite of numerous other potential health benefits of any future study remains to be seen.

Primary prevention of prostate cancer then remains a challenge, with a paucity of ongoing clinical trials. As highlighted, the design and appropriate outcome measures to balance clinically pertinent results with achievable and affordable projects is not straightforward [35, 36].

## Repurposing drugs for preventing progression in patients on active surveillance

As an alternative, and with the diagnosis of early, low risk disease rising through the increased use of PSA testing, might we be able to employ repurposed agents to treat prostate cancer at this early stage, preventing progression to situations that require radical treatment? Active surveillance is routinely offered to patients diagnosed with low-risk prostate cancer in lieu of validated biomarkers to predict progression where a significant proportion might avoid radical treatment. Active surveillance schedules must ensure comprehensive monitoring, and future strategies could use personalised deep-learning-based algorithms to predict grade reclassification [37]. A 15-year follow-up of 1643 men in the ProtecT trial (NCT02044172) found no difference in mortality between those offered active monitoring, prostatectomy or radiotherapy, however, prostatectomy and radiotherapy reduced disease progression and the development of metastases [38]. While there is no detriment to survival associated with active surveillance, there is a potential opportunity for interventions that prevent progression and hence would improve outcomes without the toxicity associated with conventional treatments. The safety net of curative treatment upon signs of progression makes men on active surveillance a unique population to trial repurposed drugs that may delay disease progression [39].

Hyperinsulinemia promotes metastasis and treatment resistance through upregulation of intracellular testosterone levels and androgen receptor activation in prostate cancer cells in vitro [40]. Some metabolic modulators, such as metformin, aim to reduce hyperinsulinemia and can influence other cancer signalling pathways due to their antineoplastic properties [41]. Originally investigated as a treatment for malaria, influenza and now type 2 diabetes [42], metformin was shown to reduce cancer incidence and cancer-specific mortality in people with diabetes compared to insulin [43] in an observational cohort study. Its mechanism of action is multifaceted, involving inhibition of cell proliferation, cell cycle progression and cellular invasion [44]. It regulates energy function in cancer stem cells (CSCs) by activating AMP-activated protein kinase (AMPK), a metabolic regulator. This inhibits oxidative phosphorylation and reduces mitochondrial ATP production [45], forcing tumour cells to increase glycolysis. Since CSCs rely heavily on oxidative phosphorylation, this creates an energy crisis that increases their sensitivity to anti-cancer treatment. The Metformin Active Surveillance (MAST) trial (NCT01864096) investigated whether metformin affected progression-free survival in men with prostate cancer on active surveillance. Eligible patients had biopsy-proven, low-risk, localized PCa (Gleason score of ≤ 6 observed in ≤ 1/3 cores, < 50% positivity in any one core, PSA ≤ 10 ng/ml, clinical stage T1c-T2a) diagnosed within the past 6 months. Patients were randomly assigned (1:1) to metformin (850 mg BID) vs. placebo for 3 years. Patients underwent repeat prostate biopsy at 18 and 36 months. Primary outcome was progression-free survival (time to primary therapy e.g., prostatectomy, radiation, hormonal therapy, or pathological progression). 141/407 patients experienced disease progression with no difference in progression-free survival (PFS) observed between patients treated with metformin and those receiving placebo (*p* = 0.63). An exploratory subgroup analysis even suggested potential detriment in patients on metformin with a higher BMI and a Gleason score of at least 8 at disease progression [46]. A meta-analysis of observational studies suggested that metformin might be of benefit as an adjuvant therapy after radical treatment [47] and as we will discuss below, trials have tested the addition of metformin in the advanced setting.

As alternative candidates, epidemiological data suggests statins may reduce the risk of advanced and fatal prostate cancers [48], and in a meta-analysis of trials of men undergoing radical treatment, patients taking statins had improved outcomes when treated with radical radiotherapy (an effect not seen in patients treated with surgery) [49, 50]. Statin prescription for cardiovascular disease is highest in men over 70 – the demographic of men with the highest incidence of prostate cancer and the significant burden of cardiovascular disease among men with prostate cancer offers a risk-benefit profile in favour of statin use. However, current observational data does not support an effect in patients on active surveillance [51] and it remains to be seen whether the community will deem a randomised controlled trial worthwhile.

Beta-blockers have also been proposed as a potential adjunct for men on active surveillance. Their cardioprotective function occurs due to inhibition of beta-adrenergic receptors, which may also have anti-neoplastic benefits as in vivo studies suggest beta-adrenergic stimulation has a role in apoptosis, angiogenesis and metastases [52]. A retrospective study showed atenolol use was associated with a reduced risk of pathologic upgrade to Gleason grade ≥ 3 [53] in men on active surveillance, but prospective trials are needed to determine causality.

While retrospective and observational studies provide suggestions of drugs to repurpose in this area, and patients on active surveillance are potentially an attractive group to study (well characterised disease, regular follow up, clear patient benefits from positive findings) clinical trials testing the efficacy of repurposed agents in men on active surveillance remain vital in producing definitive data in this setting [54]. Interestingly, although not strictly repurposed drugs in the conventional sense, there are strong parallels here with efforts to investigate dietary intervention in this setting, e.g. a high omega-3, low omega-6 diet in combination with fish oil supplementation as tested in the phase 2 CAPFISH-3 trial (NCT02176902) [55]. Given the range of possible interventions and surrogate outcome measures, there is arguably an opportunity for a multi-arm multi-stage (MAMS) trial to improve efficiency in this approach.

## Ongoing repurposing trials in the treatment of prostate cancer

### tE2 programme – improving QoL and long-term outcomes substituting tE2 for LHRHa

ADT, traditionally achieved using luteinising hormone-releasing hormone analogues (LHRHa), is central to prostate cancer management. While LHRHa effectively suppress testosterone, they also reduce circulating oestrogens which contributes to many of the significant toxicities including hot flushes, osteoporosis, increased fracture risk, adverse metabolic changes, increased cardiovascular risk and reduced quality of life. Oral oestrogens, although previously used as ADT, were abandoned due to high rates of cardiovascular thromboembolic effects linked to hepatic first-pass metabolism. Transdermal oestradiol (tE2) patches, which allow oestradiol to pass through the skin, avoid this and are licensed for hormone replacement therapy in post-menopausal women. Repurposing tE2 patches results in suppression of testosterone levels while avoiding those side-effects associated with oestrogen deficiency.

The PATCH (Prostate Adenocarcinoma Transcutaneous Hormones) repurposing programme (NCT00303784) is evaluating the repurposing of tE2 patches for ADT in men with locally advanced (M0) or metastatic (M1) prostate cancer [56]. This adaptive, multi-stage trial investigates the safety and efficacy of tE2 patches compared to LHRHa. Participants have been recruited through the PATCH and STAMPEDE (NCT00268476) trials, where a randomisation to tE2 was included as a comparison with eligibility and design aligned. Analyses will be combined, using a meta-analysis approach. This evaluation assesses tE2 patches for equivalent androgen suppression while minimising treatment-related toxicities. Importantly in this regard, the tE2 evaluation uses a non-inferiority design with respect to prostate cancer outcomes (Table 1).

Initial results from PATCH have demonstrated equivalent androgen suppression, improved quality of life, bone mineral density and superior metabolic profiles in the tE2 arm, with no significant increase in cardiovascular events (HR 1.11, 95% CI: 0.80–1.53, *p* = 0.54) [57–59]. Ultimately 2,490 patients were randomised from both trial networks, with locally advanced, non-metastatic (M0) and patients presenting with metastatic disease (M1) considered separately for the phase III analyses. The primary outcomes were metastasis-free survival (MFS) for M0 patients and overall survival (OS) for M1 patients with non-inferiority margins set to rule out a 4% detriment in 3-year MFS (HR 1.27) and 5% in OS (HR 1.19). Secondary outcomes include overall survival (for M0 patients only), progression-free survival (PFS), prostate cancer-specific survival, cardiovascular morbidity, hormone levels and quality of life (reviewed in [56]).

Results from the phase III evaluation are approaching maturity, with results presented in 2024 demonstrating that tE2 patches achieved a 3-year MFS of 87% compared to 86% with LHRHa, with a hazard ratio (HR) of 0.96 (95% CI 0.81–1.14) in favour of tE2, excluding a 2% reduction in MFS [60]. Overall survival also favoured tE2 with a HR of 0.90 (95% CI 0.75–1.07). Sustained castration rates were similar between groups (85%), with tE2 showing a reduction in patients experiencing hot flushes though with a higher incidence of gynaecomastia. Further results confirm that tE2 can safely and effectively be combined with the novel ARSI agents [61]. Final efficacy results from patients with metastatic disease (the M1 cohort) are expected in 2026, but the repurposing of tE2 potentially represents a choice for patients requiring androgen deprivation both in terms of administration and side effect profile with significant cost savings (where tE2 is currently cheaper than LHRHa). For tE2 to become widely available however, barriers remain e.g. an extension of the current licence (beyond post-menopausal hormone replacement therapy) and inclusion in national guidelines.

### Add Aspirin – a basket trial aiming to improve outcomes across multiple cancers

The analysis of RCTs investigating aspirin in the cardiovascular settings remains an exemplar of using RCT data to support repurposing. In addition to the effect on primary prevention, the analyses demonstrated a reduced risk of cancer metastasis (HR 0.64, 0.48–0.84, *P* = 0.001) [32] and the risk of metastasis on subsequent follow-up in patients without metastasis at diagnosis (hazard ratio 0.45, 0.28–0.72, *P* = 0.0009) [33] suggesting a role in secondary prevention. Add-Aspirin (NCT02804815) is a phase III, multi-centre, double-blind, placebo-controlled randomised basket trial [62] with four tumour specific cohorts of patients investigating effect of daily aspirin of 100 mg or 300 mg on disease recurrence and survival in patients who previously had completed curative-intent treatment for either breast, prostate, colorectal or upper gastrointestinal cancer [63]. The prostate cancer cohort includes men who have undergone radical treatment (surgery or radiotherapy) and are at intermediate or high risk of recurrence. The trial has a run-in period of 8 weeks where all participants take aspirin 100 mg daily to monitor tolerability and adherence to minimise drop-out in the subsequent randomised phase.

Each tumour specific cohort is individually powered. The primary outcome for the prostate cohort is biochemical recurrence-free survival (bRFS), which provides a measurable and clinically relevant indicator of treatment efficacy within a feasible timeframe, aligning with the scale and duration of the trial (Table 1). The common primary outcome for all cohorts combined is overall survival (OS). 1917 men treated for localised prostate cancer have been randomly allocated and the primary outcome analysis is expected in 2027. Long-term follow-up and the large sample size will also produce evidence of the effects of aspirin unrelated to the primary cancer, e.g. prevention of deaths related to vascular events and second malignancies. Rates of serious toxicity (especially haemorrhage), as well as other secondary health outcomes will be available. Ultimately aspirin is a low-cost drug available worldwide - if beneficial as an adjuvant treatment, even with a modest effect, it could change practice globally. A caveat to this might be in the event that any beneficial effect is only seen in a subset of patients – where additional e.g. molecular testing might be required, impacting the widespread applicability. This situation within the context of aspirin as an adjuvant treatment for colorectal cancer has been suggested by the ALASCCA trial (NCT02647099) [64] where the effect is only seen in patients with cancers containing mutations in PIK3CA.

### Repurposed drugs in STAMPEDE – investigating Celecoxib, Zoledronic acid and Metformin in men with locally advanced or metastatic prostate cancer

STAMPEDE is a multi-arm, multi-stage (MAMS) platform trial, testing the addition of treatments in the ‘upfront’ setting for patients with prostate cancer, i.e. in combination with androgen deprivation at diagnosis of hormone-sensitive locally advanced or metastatic prostate cancer. The MAMS design, highly novel when STAMPEDE opened in 2006, allows for efficient assessment of multiple interventions while maintaining statistical power [65]. Positive findings from STAMPEDE have included the benefits of the addition of upfront Docetaxel and Abiraterone, and improvements in outcomes for patients with low volume metastatic disease who receive radiotherapy to the prostate [12, 13, 66]. With respect to repurposed agents, Celecoxib, Zoledronic acid and Metformin have all been investigated within STAMPEDE with the primary aim of improving cancer outcomes, targeting an improvement in overall survival with a superiority design.

Celecoxib, a selective cyclo-oxygenase 2 (COX-2) non-steroidal anti-inflammatory drug (NSAID), and Zoledronic acid, a bisphosphonate, were assessed in the STAMPEDE trial, alone and in combination, with patients recruited between 2005 and 2011. COX-2 is thought to have roles in immune evasion and promoting inflammatory pathways that promote tumourigenesis - inhibition therefore facilitating immune recognition [67]. Zoledronic acid inhibits the mevalonate pathway potentially inducing apoptosis in prostate cancer cells and disrupting the bone microenvironment to reduce tumour colonisation and osteoclast-mediated bone destruction [68]. A total of 1,245 patients were randomised across three arms: Arm 1 (SOC, 622 patients), Arm 2 (SOC + Celecoxib, 312 patients), and Arm 3 (SOC + Celecoxib + Zoledronic acid, 311 patients). SOC was androgen suppression either continuously (metastatic) or for ≥ 2 years (non-metastatic). Radiotherapy to the prostate (+/- pelvic nodes) was recommended for patients without distant metastasis. Treatment durations were Celecoxib for 1 year (400 mg PO BD) and Zoledronic acid 4 mg, for six cycles every 3 weeks then 4-weekly for 2 years.

Treatment was well-tolerated across all three arms, with similar G3-5 adverse event rates (36% in SOC-only, 33% in Celecoxib + SOC, and 32% in Celecoxib + Zoledronic acid + SOC). Osteonecrosis of the jaw was reported in 6 cases (2%) in arm 3 [69].

Celecoxib containing arms were discontinued for accrual due to failing to meet pre-planned activity criteria based on failure free survival (FFS), primarily driven by rising PSA. Subsequent follow up demonstrated no evidence of a benefit to overall survival [69]. Similarly, across the whole cohort (non-metastatic and metastatic patients) there was no benefit to the combination of Celecoxib and Zoledronic Acid (overall survival HR 0.86 (95% CI, 0.70 to 1.05; *p* = 0.130)). However, in pre-planned analyses of the metastatic patients (*n* = 755), the combination improved both overall survival (HR 0.78; 95% CI, 0.62 to 0.98; *p* = 0.033) and prostate cancer-specific survival (HR 0.64 (95% CI, 0.49 to 0.83)). Although insufficient data to change practice, this finding certainly supports further study of this combination in patients with metastatic disease.

As discussed above, there is a strong pre-clinical rationale for testing metformin in patients with prostate cancer, and the STAMPEDE platform provided the ideal platform to do this. 1874 patients with metastatic prostate cancer were randomised between standard of care (SOC) or SOC + Metformin (850 mg BD, lifelong if tolerated). Metformin did not significantly improve overall survival (the primary outcome measure, (HR 0.91, *P* = 0.1479) [70]. In patients with the highest risk disease (high versus low-volume disease defined as per CHAARTED) [11], the HR for metformin arm vs. SOC was 0.79 (95% CI 0.67–0.94) in the high-risk and 0.98 (95% CI 0.78–1.23) in the low-risk patients(*p* = 0.1206). Morphological and metabolic outcomes (including change in weight, waist circumference, change in glucose, and lipid profile) were statistically better on the Metformin arm. Similar to the findings with respect to Celecoxib-Zoledronic acid, although the trial did not meet its primary outcome measure across the whole population the results seen in the metastatic patients is provocative, with apparent benefits (oncological and metabolic) in specific sub-groups. Whilst not enough to recommend a change in practice at this point, it provides support for further study. In particular further translational research is planned to try to identify subgroups who gain most benefit from the addition of metformin.

## Conclusions

Prostate cancer is a significant and growing global health challenge, both in terms of numbers of patients and cost to the world economy. Repurposed agents offer an opportunity to improve outcomes for patients and are being tested at each point of the disease from prevention to the advanced setting and the list of drugs with potential benefits continues to grow [71]. However, to date, results from adequately powered phase III trials reflect the challenges associated with this approach and only tE2 has demonstrated clinically meaningful efficacy with benefits that support it as a choice for men with prostate cancer. It is noteworthy that with tE2 patches the mode of action (androgen suppression) was well understood prior to the trial. In contrast, although many mechanisms have been proposed for the wide range of potential repurposed agents, trials have generally progressed ahead of this knowledge given that the drugs are well tolerated and relatively inexpensive. It could be argued that for the field to really progress these gaps need addressing – well tolerated and inexpensive with some supportive observational data is not enough. Ultimately this is likely to require much better understanding of the underlying science and predictive biomarkers - as demonstrated in the case of aspirin and PIK3CA mutations in the context of colorectal cancer in the ALASCCA trial [64]. It has also been suggested that academia – where most of the repurposing efforts take place – learn from industry approaches such as the Target Product Profile) [72, 73].

An important consideration for repurposed agents, assuming ultimately the data supports their use, is in the subsequent licensing for this new indication. This has implications not just for the treating clinician but goes back to trial design and conduct where it is advisable that triallists work with the relevant regulatory authorities from the outset on repurposing projects [74]. One precedent in this area is Anastrozole for the prevention of breast cancer in women at high risk of the disease [75], which has had its licence extended for this indication over and above its initial development for the treatment of established disease. Challenges remain in this situation for the predominantly academic community who undertake the repurposing projects as typically patents have long since expired and pharmaceutical company support, often a prerequisite for changes to license, may be lacking. Initiatives aiming to address issues relating to repurposing (including on licence extensions) could be undertaken by governments in particular [76]. LifeArc has developed a medicine repurposing toolkit to support academic team in their repurposing journey. REMEDI4ALL, the European platform for medicines repurposing, supports researchers and investigators across the whole drug repurposing path (from candidate identification to licence extension and reimbursement).

Conceptually, repurposing drugs for the treatment of patients with prostate cancer holds significant promise, with a wealth of potential agents demonstrating biologically plausible mechanisms and supporting pre-clinical and observational data. Similarly, the safety profile of repurposing candidates is often well understood which is a major advantage over novel agents. Ultimately the bar to becoming standard of care remains as high as for new drugs, and there are no short-cuts to the quality of clinical data required to satisfy regulators, clinicians and patients alike. Currently there remains a paucity of examples of drugs successfully repurposed for cancer. As the trials discussed above reach their primary analyses, it is hoped that this may change with significant benefits in terms of patient and wider health economic outcomes.

## Data Availability

No datasets were generated or analysed during the current study.

## Abbreviations

ADT: Androgen Deprivation Therapy
ARSI: Androgen Receptor Signalling Inhibitor
bPFS: Biochemical Progression Free Survival
CSC: Cancer Stem Cells
COX1: Cyclo-oxygenase 1
COX2: Cyclo-oxygenase 2
dMMR: deficient Mismatch Repair
FFS: Failure Free Survival
HR: Hazard Ratio
LHRHa: Luteinising Hormone Releasing Hormone agonist
MAMS: Multi-Arm Multi-Stage
MFS: Metastases Free Survival
OS: Overall Survival
PARP: Poly ADP Ribose
PSA: Prostate Specific Antigen
PSMA: Prostate Specific Membrane Antigen
SOC: Standard of Care
tE2: transdermal Estradiol
